# Fantastic genes and where to find them expressed in CHO

**DOI:** 10.1016/j.csbj.2025.03.050

**Published:** 2025-04-02

**Authors:** Markus Riedl, Caterina Ruggeri, Nicolas Marx, Nicole Borth

**Affiliations:** Department of Biotechnology, BOKU University, Vienna, Austria

**Keywords:** Chinese hamster ovary cells, Transcriptomics, Meta-analysis, Gene expression

## Abstract

The transcriptome of Chinese hamster ovary (CHO) cells plays a crucial role in determining cellular characteristics that are essential for biopharmaceutical applications. RNA-sequencing has been extensively used to profile gene expression patterns, aiming to gain a better understanding of intracellular behavior and mechanisms. Individual datasets, however, do not provide a comprehensive overview and characterization of the CHO cell's transcriptome, such that the fundamental structure of the transcriptome remains unknown. Using 15 RNA-sequencing datasets, encompassing almost 300 samples of various experimental setups, conditions and cell lines, we explore and classify the protein-coding transcriptome of CHO cells. Differences in cell line lineages are found to be the primary source of variation in transcribed genes. By employing a novel approach, we identified the core transcriptome that is ubiquitously expressed in all cell lines and culture conditions, as well as genes that remain entirely non-expressed. Additionally, we identified a set of genes that may be active or inactive depending on different conditions, which are linked to biological processes including translation as well as immune and stress response. Lastly, by integrating chromatin states derived from histone modifications, we provided additional context on the epigenetic level that supports our protein-coding gene classification. Our study offers a comprehensive insight into the CHO cell transcriptome and lays the foundation for future research into cellular adaptation to changing conditions and the development of phenotypes.

## Highlights

• Expressed and non-expressed genes are highly consistent across all CHO cell lines.
• CHO lineages are the primary factor of variation in transcribed genes in CHO.
• A subset of genes exhibits large shifts in expression level, potentially in response to changing environmental conditions.
• Chromatin states validate expression gene sets and provide insight into epigenetic regulation.

## Graphical abstract

**Figure:**
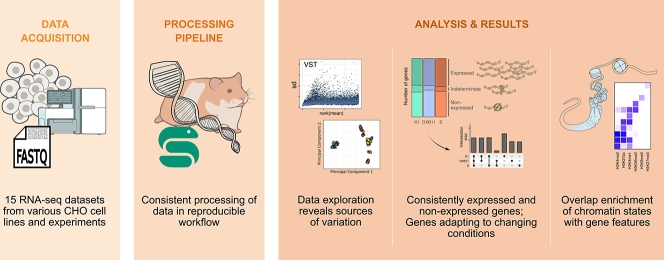

## 1 Introduction

Chinese Hamster Ovary (CHO) cells are a pillar of pharmaceutical biotechnology. As the main cell factory to manufacture complex biological therapeutics, notably monoclonal antibodies, they produce a significant portion of the highest-selling pharmaceuticals globally [1], [2]. Since their initial isolation in the 1950s [3], CHO cells have become indispensable within the biopharmaceutical industry. Numerous advantages set them apart from other production systems, including their ability to proliferate in suspension cultures in chemically defined media, achieving high viable cell densities. Additionally, CHO cells exhibit glycosylation patterns akin to human cells when producing recombinant proteins, a critical quality attribute for therapeutic applications [4], [5], [6]. Overall, CHO cells ensure a high degree of product yield and quality while being easier to cultivate than other mammalian cell lines, justifying their continued use in biotechnology [7].

Efforts to improve attributes relating to CHO cells as a production platform further augmented their status as the workhorse for manufacturing of complex biotherapeutics. Over time and in consequence of different treatments such as mutagenesis, clonal selection and, more recently, genome editing, different lineages of CHO have been established, including CHO-K1, CHO-DXB11, CHO-S and CHO-DG44, each of which exhibits distinct characteristics and properties relating to growth, productivity and cell selection strategies, among others [7]. In recent years, novel genetic engineering tools have further empowered researchers with unprecedented precision for targeted cellular engineering [8], [9], [10], [11]. Any such endeavors to improve CHO cells are aided by—and may even require—a profound understanding of the fundamental mechanisms that govern cellular phenotypes. A particularly useful approach includes the use of omics methods that allow for systematic investigations of intracellular mechanisms on several levels such as the genome, epigenome, transcriptome or proteome.

Genomic studies in CHO cells revealed highly unstable genomes with numerous mutations, structural variations and karyotypic rearrangements [12], [13]. Many such genomic variations were found to be unique to certain cell lines or cell line lineages [14], [15]. Interestingly, despite the genomic disparities between CHO cells and their originating organism, the Chinese hamster, the overall size of the genome remains comparable, with most regions of the Chinese hamster genome still present in the different cell lines [12], [16]. On top of the genetic heterogeneity in CHO cells, profiling of the epigenetic control of gene expression revealed highly dynamic processes of adaptation to different conditions and growth phases [17], [18]. Eventually, the phenotype of a cell is largely determined by the transcriptome and, consequently, its gene products. Thus, differential analysis of the transcriptome between conditions is a highly informative approach to profile cellular behavior. RNA-sequencing (RNA-seq) is most often used to analyze differentially expressed genes and has become a common practice towards understanding and manipulating phenotypes of CHO cells. This approach was instrumental in investigating gene expression changes following temperature shift [19], [20], adaptation to glutamine-free media [21], subcloning procedures [22], [23], as well as in uncovering disparities between high- and low-producer phenotypes [24], and the impact of viral infection on CHO cells [25], [26], among other research questions.

Although RNA-seq studies are valuable for identifying differentially expressed genes under variable conditions, individual RNA-seq experiments for specific CHO cell lines are a limited resource for a comprehensive overview and understanding of the general CHO transcriptome. The significant differences in genotype and karyotype between lineages as well as the epigenetic dynamics that influence gene expression may limit transferability of results across lineages [10]. Importantly, these genetic and epigenetic effects are not only observed when comparing cell lines of different lineages but also for the same cell line over prolonged time in culture or in changing culture conditions [17], [18], which may also limit the transferability across individual experiments of the same lineage. Consequently, there remains a need for a detailed description of the core CHO transcriptome that defines a universal CHO cell. Furthermore, it remains unknown to which extent phenotypic differences and responses to changing culture conditions are the product of absolute activation or repression of the expression of individual genes or whether it is due to intricate, fine-tuned control of gene expression levels across thousands of expressed genes. Such knowledge would greatly enhance the interpretation of transcriptome studies in CHO cells and would allow for better transferability of genotype-to-phenotype experiments.

With numerous RNA-seq datasets available in the public domain, we here present a meta-analysis of the CHO coding transcriptome across various cell lines, culture conditions and phenotypes. Using a novel approach, we categorize the expression of all protein-coding genes in the CHO genome and delineate differences and similarities across cell lines. In addition, we identify genes that are activated or repressed in response to different conditions and therefore exhibit a large change in expression level. Our findings are further contextualized with additional omics data, providing insights into mechanisms regulating gene expression. This work underscores the power of utilizing public data resources to achieve a comprehensive understanding of intracellular mechanisms across the diversity of cell lines and culture conditions. The findings published here advance the understanding of the fundamental transcriptomic patterns underlying CHO cell characteristics.

## 2 Materials and methods

### 2.1 Data acquisition

RNA-seq datasets were acquired from the Sequence Read Archive (SRA) of the National Center for Biotechnology Information (NCBI) and converted to FASTQ using the prefetch and fasterq-dump utilities from SRA Toolkit v2.11.0. In-house datasets stored as BAM files were converted to FASTQ using bamToFastq from bedtools v2.30.0.

### 2.2 RNA-seq reads processing

All RNA-seq raw data was consistently processed through a custom-developed and fully reproducible workflow using the workflow management software Snakemake [27]. All samples were processed by the workflow in consideration of their technical and methodological properties including single- and paired-end reads, unstranded and forward/reverse stranded reads as well as presence of technical replicates and different sequencing adapters.

Raw reads in FASTQ format were trimmed with trimmomatic v0.39 [28]. Trimming criteria included sequencing adapters, a minimum read length of 36 bases and a quality-based threshold at a phred score of 15 for leading and trailing bases as well as in a sliding window of four bases. FastQC v0.12.1 [29] was used to inspect reads before and after trimming. Trimmed reads were aligned to the PICRH genome (NCBI RefSeq assembly GCF_003668045.3 and annotation release 104; downloaded on May, 17 2023) [30] using STAR v2.7.10b [31]. The strandedness of aligned reads was estimated using the infer_experiment utility from RSeQC v5.0.1 [32]. Reads were quantified on gene level using the featureCounts utility from Subread v2.0.1 [33].

### 2.3 Data exploration

Read counts derived from featureCounts were read into R v4.3.3 [34]. Prior to the transformation, genes with low read counts were filtered using the approach recommended by Chen et al. [35]. Briefly, the approach filters out genes with less than approximately 10 counts of a gene in at least the 20 samples, the average number of samples in the included datasets. To make the filtering step more robust against large differences in library size, Counts Per Million (CPM) were used instead of raw counts. The CPM threshold of 0.5 corresponds to 10 counts at a library size of 20 M reads. Regularized logarithm transformation or variance-stabilizing transformation were applied to filtered gene counts using the rlog or vst functions, respectively, from the R package DESeq2 v1.42.0 [36]. These functions include library size normalization using the median of ratios method. Principal Component Analysis (PCA) was done using the plotPCA function from DESeq2; Principal Coordinate Analysis (PCoA) was conducted using the plotMDS function from edgeR v4.0.16 [37]. Both, PCA and PCoA were conducted on the 500 most variable genes.

### 2.4 Stratification of genes

The unfiltered raw gene counts matrix from featureCounts was subset to contain only protein-coding genes according to the GTF annotation of the PICRH genome assembly. The normalization of the read counts was performed according to the Gene length corrected Trimmed Means of M-values (GeTMM) approach by Smid et al. [38] to normalize for the comparison between and within the samples. Briefly, raw gene counts are corrected by gene length by calculating the number of reads per kilobase of the longest transcript, i.e. the sum of exon lengths of the longest transcript of a gene according to the genome annotation. TMM normalization factors were computed using the edgeR function calcNormFactors. Finally, log_2_CPM values were computed from gene-length corrected read counts with a prior count of 0.25 as well as the normalized library sizes and used for subsequent analyses.

Coding genes were stratified into expressed and non-expressed based on a normalized log_2_CPM cutoff of 0. They were considered expressed in a cell line if their log_2_CPM values were >0 in >95% of samples. Conversely, genes were classified as non-expressed in a cell line if the log_2_CPM values were <0 in >95% of samples. Genes that were neither consistently expressed nor non-expressed in all studied samples of a cell line were classified as indeterminate. In order to further distinguish indeterminate genes expressed around the threshold from genes that react to different culture conditions, the latter termed reactive genes, Hartigan's dip test [39] was applied to all gene expression distributions in each cell line using the R package diptest v0.77-1. Reactive genes were defined based on a Benjamini-Hochberg [40] adjusted p<0.05.

### 2.5 Functional enrichment

Over-representation analysis of Gene Ontology (GO) biological process terms of all gene sets was done using clusterProfiler v4.10.0 [41] which uses a one-sided Fisher's exact test for hypothesis testing. AnnotationHub v3.10.0 was used to retrieve the annotation database for *Cricetulus griseus* (AH114610) with snapshot date 2023-10-21. KEGG pathways were similarly enriched on all gene sets with clusterProfiler using the KEGG organism databases ‘cge’. Multiple testing correction by Benjamini-Hochberg [40] was employed with an adjusted *p*-value cutoff of 0.05. Significantly enriched GO biological process terms were simplified to remove redundancy using a semantic similarity (Wang method [42]) cutoff of 0.7.

### 2.6 Chromatin states enrichment

Chromatin states were predicted from a previously published ChIP-seq dataset by Feichtinger et al. [17]. Briefly, CHO-K1 (ECACC CCL-61) adapted to protein-free suspension medium was cultivated in a batch culture for 9 days and sampled every 12 hours. ChIP-seq data included histone marks H3K4me3, H3K4me1, H3K9me3, H3K27ac, H3K27me3 and H3K36me3 for each time point. Chromatin states were learned from histone marks for the PICRH genome assembly using ChromHMM v1.24 [43] as described by Feichtinger et al. [17] and presented in Rupp et al. [44].

For the calculation of chromatin state enrichments, the PICRH genome annotation was filtered into subsets containing only the previously determined expressed, reactive and non-expressed protein-coding genes of CHO-K1, respectively. From these annotation subsets, the coordinates of gene bodies, exons, Transcript Start Site (TSS), Transcript End Site (TES) as well as a window of 2 kbp upstream and downstream of the TSS were extracted. Chromatin state overlap enrichment was performed on the genomic regions (gene bodies, exons and TSS ± 2 kbp), neighborhood enrichment on TSS and TES positions. Enrichments were calculated from the chromatin state posteriors for each annotation using the OverlapEnrichment or NeighborhoodEnrichment command of ChromHMM. Enrichment values were subsequently scaled to the interval [0,1].

## 3 Results and discussion

### 3.1 Description of included datasets

**Fig. 1:**
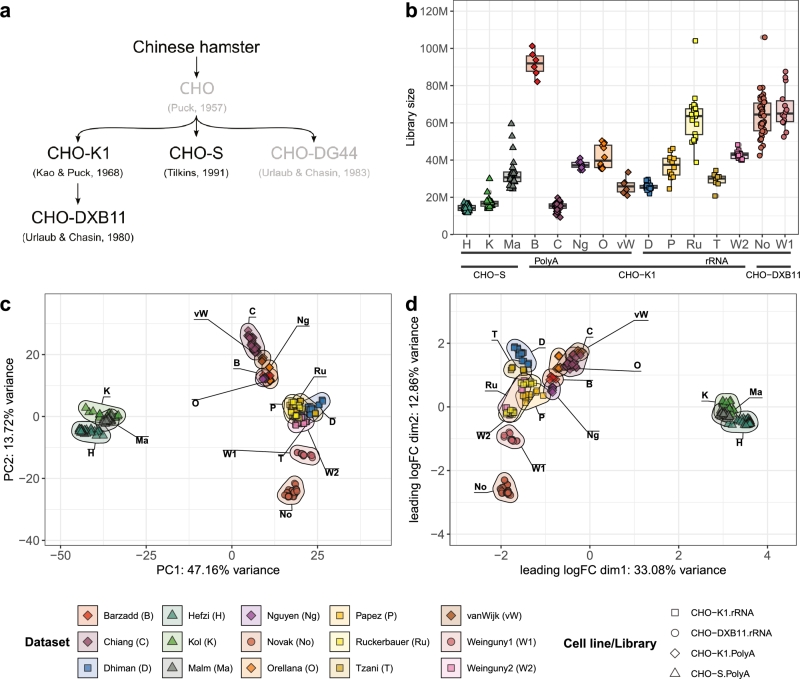
Data exploration of RNA-seq samples from 15 datasets. Samples are colored by datasets, shapes represent the cell line and library preparation method. **(a)** Simplified family tree of CHO cell lineages. Cell lines not included in our study are grayed out. Adapted from Lewis et al. [12]. **(b)** Boxplot of library sizes of included samples. **(c)** Principal component analysis from variance-stabilizing transformed counts. Samples from individual datasets are marked and labeled with the corresponding dataset's abbreviation. **(d)** Principal coordinate analysis from GeTMM normalized logCPM. Samples from individual datasets are marked and labeled with the corresponding dataset's abbreviation.

We selected 15 RNA-seq datasets amounting to 293 individual samples encompassing various different biological phenotypes and culture conditions. Ten datasets were from CHO-K1 cell lines (samples n=153), three from CHO-S (n=84) and two from CHO-DXB11 (n=56). The datasets differ in technical aspects such as single- and paired-end sequencing (samples n=102 and 191, respectively) as well as library preparation methods poly(A) enrichment and rRNA depletion (n=154 and 139). The datasets and some of their technical and biological characteristics are listed in Table 1. An extensive description of the included datasets and samples is given in Supplementary File 2. Among the cell lines used in the different studies are both strains from standardized cell culture collections ATCC [51] and ECACC [52] as well as commercial expression systems such as CHOZN® GS-/- (Sigma-Aldrich, St. Louis, USA), Horizon Discovery CHO-K1 GS-/- (Horizon Discovery, Cambridge, UK) or Freestyle™ CHO-S (Thermo-Fisher Scientific Inc., Waltham, USA). In order to streamline the analysis, all cell lines were assigned to their respective cell line of origin (i.e. CHO-K1, CHO-DXB11 or CHO-S). Fig. 1a shows the relationships of the three cell lines in our study.

**Table 1:** List of RNA-seq datasets used in the meta-analysis with information on cell line, process conditions and methods used. Full details of cell lines, their origin and properties, as reported in the original publications, are provided in Supplementary File 2.

	Author	Cell line	Library type^1^	Description	Ref.^2^
B	Barzadd	K1	PE, poly(A)	Reduction of bispecific antibody aggregation.	[45]
C	Chiang	K1	PE, poly(A)	Characterization and improvement of viral resistance.	[25]
D	Dhiman	K1	PE, rRNA	Expression stability characterization of transgenes.	[46]
H	Hefzi	S	PE, poly(A)	Consensus genome-scale metabolic model.	[47]
K	Kol	S	PE, poly(A)	Host cell protein knock-outs for favorable characteristics.	[48]
Ma	Malm	S	PE, poly(A)	Secretory pathway differences between CHO and HEK293.	[49]
Ng	Nguyen	K1	SE, poly(A)	Identification of cold-shock induced genes and promoters.	[19]
No	Novak	DXB11	SE, rRNA	Analysis of lncRNAs that correlate with productivity.	[50]
O	Orellana	K1	PE, poly(A)	Differences between high and low producing clones.	[24]
P	Papež	K1	PE, rRNA	Early transcriptomic response to glutamine deprivation.	[21]
Ru	Ruckerbauer	K1	SE, rRNA	Steady-state at different growth rates.	n.p.
T	Tzani	K1	PE, rRNA	Effects of temperature shift on the transcriptome.	[20]
vW	van Wijk	K1	PE, poly(A)	Investigating factors for bacterial invasion.	[26]
W1	Weinguny	DXB11	SE, rRNA	Profiling changes upon subcloning procedures.	[22]
W2	Weinguny	K1	SE, rRNA	Directed evolution approach using rapid outgrowth.	[23]

1 SE, Single-end; PE, Paired-end.

2 n.p., not published.

### 3.2 Data exploration reveals biological variation among CHO cells

First, we inspected the library sizes of included datasets and observed a large range of library sizes, from 9.24 M to 106 M reads, with an average library size of 37.1 M reads (SD = 22 M) (Fig. 1b). For data exploration, we filtered out low expressed genes, yielding 13,121 remaining out of 21,841 protein-coding genes. Gene counts were then transformed using variance-stabilizing transformation to investigate the sources of variation among the included datasets using PCA. The first principal component explains 47.16% of total variation and can largely be attributed to the differences between the cell line CHO-S and the two closely related cell lines CHO-K1 and CHO-DXB11 (Fig. 1c and 1a). Findings by Singh et al. [53] have previously identified cell line lineages as the largest effect of differentiation in their study. Their RNA-seq datasets, one for CHO-K1, CHO-S and CHO-DG44 each, originated from different laboratories, however, which potentially leads to the confounding influence of inter-lab variability. Here, with several datasets of each cell line under investigation, originating from different laboratories and experiments, we can confirm the large differentiating effect between cell line lineages.

A second large factor that drives differentiation among RNA-seq samples, visible in CHO-K1 datasets in our study, are the library preparation strategies of poly(A) enrichment (samples n=154) and rRNA depletion (n=139). Poly(A) enrichment selects transcripts with poly-adenylated tails which includes mRNA and some non-coding RNAs, while rRNA depletion retains also non-adenylated transcripts, but simply removes rRNA. Even though protein-coding genes are preserved in both methods, we observed large differences resulting from the choice of library preparation method with a clear separation in CHO-K1 datasets based on their respective library preparation method (Fig. 1c). This differentiation is likely due to systematic differences in RNA composition after rRNA depletion or poly(A) enrichment that have already been described in literature [54], [55]. The use of single- or paired-end sequencing technology did not yield large differences between samples (Fig. S1a; n=102 and 191 for single- and paired-end sequencing, respectively). In single-end sequencing, every fragment is read once, producing one read, while in paired-end sequencing every fragment is read from both ends resulting in two reads that facilitate a more accurate mapping to the genome or transcriptome. The application of these methods depends on the requirements of the individual experiment and is largely driven by considerations of cost efficiency. The two methods can produce different results in gene counts [56] which, however, does not appear to be a large factor in our meta-analysis. In addition, the development of stranded sequencing of transcripts provided large benefits to applications such as differential gene expression [57]. In our study, 287 samples used stranded RNA-seq libraries while 6 samples used unstranded libraries.

The original CHO-K1 cell line is adherent and now oftentimes adapted to grow in suspension. Only the studies by Chiang et al. [25] and van Wijk et al. [26] used adherent cultivation of CHO-K1 in cell culture plates which visibly sets these RNA-seq samples apart from suspension-adapted CHO-K1 cultivation of other studies (Fig. 1c). This indicates that adaptation of CHO cells to suspension culture induces major changes in the transcriptome. The transcriptomic changes following adaptation to suspension growth in protein-free media have been previously described by Shridhar et al. [58] using microarray technology.

The two CHO-DXB11 datasets from Weinguny (W1) [22] and Novak (No) [50] are considerably differentiated despite being produced in the same lab and both using rRNA depletion. This difference might be explained by the distinct origin of the cell lines these studies used. The CHO-DXB11 cell line used by Weinguny was established by Lattenmayer et al. [59] from the ATCC host cell line CRL-9096 [60] while the cell line used by Novak is a proprietary cell line derived from CHO-DXB11 (Sanofi, Cambridge, USA). Therefore, these two cell lines have significantly different histories, with different adaptation and selection protocols in place, which explains the large difference between the two datasets.

To investigate the effect of different normalization and transformation methods for data exploration in our meta-analysis, we repeated the PCA using regularized logarithm transformation as well as GeTMM normalization with an unregularized log_2_ transformation. Regularized logarithm transformation was described to be more stable for highly variable library sizes compared to variance-stabilizing transformation [36]. While the transformation successfully rendered the data homoskedastic (Fig. S1c), it struggled with two datasets that have a very low library size (Fig. 1b, C and K datasets) and led to a high noise in such datasets, visible in PCA (Fig. S1d). This transformation method was therefore not adequate for data exploration in our meta-analysis. The unregularized log_2_ transformation of GeTMM normalized CPM values overinflated the variance at low expression level (Fig. S1c). The resulting PCA plot, however, yielded a highly similar structure of the data compared to variance-stabilizing transformation based PCA (Fig. S1d). A recent publication by Bairakdar et al. [61] also used GeTMM as their normalization method, in combination with batch correction methods, for quantitative machine learning applications. They reported large inter-lab variability that impeded their analysis, even after normalization and batch correction. Among our samples, inter-lab variability did not seem to play a major role as datasets from different labs were highly overlapping. Variance could mainly be attributed to the aforementioned biological and technical factors such as cell line and library preparation method.

Alternatively to PCA, Principal Coordinate Analysis (PCoA) can be applied for data visualization, using the root-mean-square of leading log_2_ fold changes between each pair of samples. As in PCA, datasets are separated based on lineage, although less distinctly by the employed library preparation method (Fig. 1d). This analysis further makes different experimental conditions and cell factories clearly visible. The datasets from Weinguny (W2) [23] and Ruckerbauer (Ru) are highly overlapping and both used CHO-K1 from ECACC as well as the CHO-K1 Hy expression system in their studies, which are visibly separated from one another indicating that this analysis reliably captures biological differences.

### 3.3 Expression-based stratification of genes reveals the core transcriptome of CHO

To elucidate the expression of genes in each cell line, the raw counts were normalized for within- and across-sample comparison using GeTMM. We investigated the effect of the normalization on three samples, representative of the whole range of library sizes (15, 38 and 94 million reads, respectively). Samples with a large library size were able to detect genes with very low expression, while with a small library size, these same genes had zero counts (Fig. S2). At higher expression of genes, the distribution of normalized log_2_CPM was nearly identical after normalization. Based on this observation we chose a log_2_CPM expression threshold of 0 to classify genes as expressed, non-expressed or indeterminate for each cell line. Additionally, we chose to use a threshold of 95% of samples per cell line to define the respective classes of expression, to reduce possible influences of individual outliers. Therefore, genes with an expression greater than 0 log_2_CPM in more than 95% of samples of a cell line were classified as expressed in that cell line. Conversely, genes with an expression less than 0 log_2_CPM in more than 95% of samples of a cell line were classified as non-expressed. The remaining genes, that were neither expressed nor non-expressed, were classified as indeterminate. It should be noted that, due to the large range of library sizes of the included RNA-seq samples (Fig. 1b), a clear differentiation of truly non-expressed genes and very lowly expressed genes is not possible. The resulting classifications of all protein-coding genes in each cell line are available in Supplementary File 3.

**Fig. 2:**
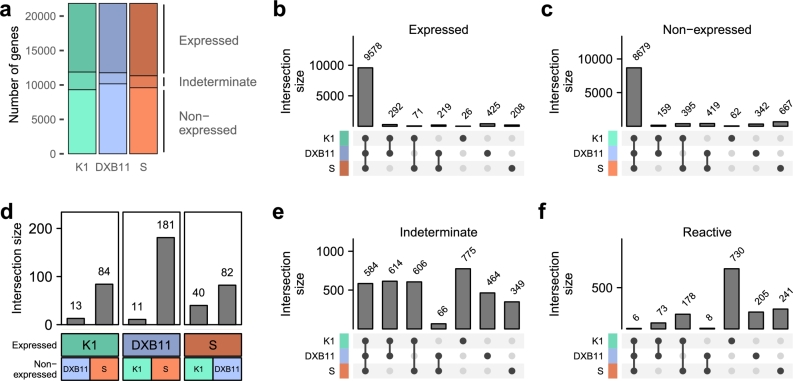
Stratification of protein-coding genes according to expression state. **(a)** Distribution of expression classes across cell lines. **(b,c,e,f)** Upset plots showing distinct intersections of (b) expressed, (c) non-expressed, (e) indeterminate and (f) reactive genes across cell lines. **(d)** Number of intersecting genes between expressed and non-expressed gene sets of cell lines.

Similar numbers of protein-coding genes were found expressed in the three cell lines (Fig. 2a), with 10,819 expressed protein-coding genes in at least one of the three cell lines. Of those, 9,578 (88.53%) were consistently expressed across all cell lines (Fig. 2b). Genes that were expressed exclusively in one or two cell lines were only in the range of tens to hundreds. On the other hand, 8,679 genes were found to be non-expressed in all cell lines and conditions investigated (Fig. 2c). Only a small number of genes were exclusively non-expressed in one or two cell lines.

Differences between cell lines were observed by comparing gene sets of expressed genes in one cell line to non-expressed genes in other cell lines. The largest intersection of 181 genes was seen in expressed genes in CHO-DXB11 and non-expressed genes in CHO-S (Fig. 2d), the smallest intersection between CHO-K1 and CHO-DXB11, with only 13 and 11 genes expressed in CHO-K1 but not in CHO-DXB11 and vice versa. This reflects the relative relatedness among the investigated cell lines, since CHO-DXB11 is a descendant of CHO-K1 while CHO-S is a different lineage stemming from the CHO cell line by Puck et al. [62].

The third expression class, termed indeterminate, at which genes were either expressed at a low level around the minimum expression threshold or only expressed in a subset of samples of a cell line, was the highest in CHO-K1 with 2,579 genes, compared to 1,605 in CHO-S and 1,728 in CHO-DXB11 (Fig. 2e). The largest number of datasets, and therefore also culture conditions and phenotypes, were from CHO-K1, which might explain the increased gene expression variability. A previous study, however, also found CHO-K1 to have the highest variability compared to other cell lines [53].

We next examined the influence of the library preparation methods on the classification of gene expression in CHO-K1 (in other cell lines only one of these methods was represented in datasets; see Table 1) and found minor differences between poly(A) enriched and rRNA depleted samples (Fig. S3). Generally, poly(A) enrichment yielded slightly more expressed and indeterminate genes than rRNA depletion (120 and 158 more genes, respectively), while the latter yielded more non-expressed genes (278 more). This systematic discrepancy is likely due to differences in the resulting RNA composition between the two RNA selection methods. Such discrepancies have been described in previous studies [54], [55].

### 3.4 Assessing the variability of indeterminate genes

Indeterminate genes are neither consistently expressed nor non-expressed. This group is composed of genes expressed at or around the expression threshold of 0 log_2_CPM, and of genes that react to changing conditions by a major shift in expression levels, either activation or repression of gene expression. The latter, hereinafter referred to as reactive genes, exhibit a very low expression below the expression threshold in some conditions and a high expression above the threshold in others. It should be noted that, in this context, reactive genes refer specifically to those within the indeterminate gene set. Unlike expressed genes that respond to changing conditions, they are oftentimes not considered in quantitative differential gene expression of RNA-seq since, depending on the implementation, genes with a low expression in a number of samples are filtered out to mitigate the implications of noise in these measurements. Therefore, there is interest in quantifying this highly dynamic group of genes that might have previously been neglected. It should further be noted that, expressed genes that respond to changing conditions and perturbations by altered expression levels are best identified using the aforementioned quantitative differential gene expression experiments.

In order to distinguish the two types of indeterminate genes, we assumed that genes with rather constant expression around the set threshold will more likely exhibit a unimodal distribution whereas genes that react to conditions will have a bi- or multimodal distribution of normalized gene expression. We therefore applied Hartigan's dip test for unimodal distributions to find and define reactive genes from all gene expression distributions of indeterminate genes.

In total, 1,441 genes were found to be non-unimodal in their gene expression distribution (Fig. 2f). Of those, the most were found in CHO-K1 with 987 genes which is likely due to the high number of datasets with different experiments, compared to the other cell lines in our analysis. From CHO-S, 433 were defined as reactive genes and from CHO-DXB11 292 genes. The concordance in the number of experiments and the number of reactive genes in the cell lines suggests that the dip test reliably separates genes that are expressed at or around the threshold from genes that react to different culture conditions. Furthermore, subsetting the indeterminate genes to reactive genes greatly reduces the number of intersecting genes between cell lines, supporting this conclusion (Fig. 2e and 2f).

### 3.5 Functional enrichment of stratified gene sets

We next wanted to investigate whether there are significantly enriched biological processes in these previously determined gene sets. Therefore, we conducted over-representation analysis of GO biological process and KEGG pathway terms on all gene sets of expressed, non-expressed and reactive genes as well as all genes that were expressed exclusively or differentially between cell lines. For GO analyses, 4,946 protein-coding genes were annotated with GO biological process terms and used as background for the over-representation analysis. Three gene sets of differentially expressed genes, i.e. expressed in one but non-expressed in another cell line, were enriched in GO biological process terms (Table S1). However, the over-representation of biological process terms is based on only one to three genes in these gene sets, hence these differences are likely not meaningful in terms of biological processes.

**Fig. 3:**
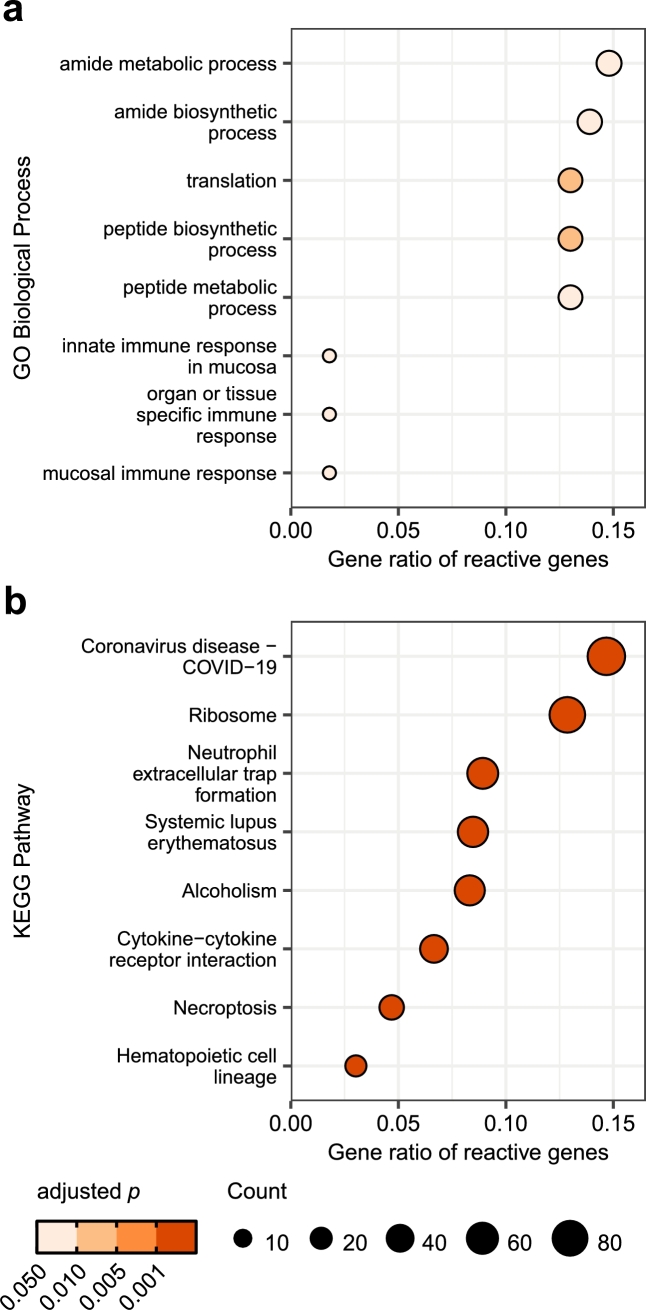
Biological context of genes classified as reactive in at least one cell line. **(a)** GO biological process terms and **(b)** KEGG pathway terms significantly enriched in reactive genes. Adjustment of *p*-values was done using Benjamini-Hochberg correction. GO, Gene Ontology; KEGG, Kyoto Encyclopedia of Genes and Genomes.

More interestingly, the gene set of reactive genes was highly enriched in genes relating to translation and peptide biosynthesis, visible in GO biological processes (Fig. 3a) as well as KEGG pathways (Fig. 3b). The genes associated with the KEGG pathway ‘Ribosome’ (cge03010, $p=1.02\times 10^{-26}$) consist of both ribosomal proteins as well as ribosomal like proteins of 60S and 40S subunits (Fig. S4). In addition to their role in protein biosynthesis, ribosomal proteins, and ribosomal like proteins, play an important role in stress response mechanisms and maintenance of genomic integrity [63], [64], [65]. Therefore, the changes in gene expression might be induced as stress response in different culture conditions. The enrichment of the KEGG pathway ‘Coronavirus disease - COVID-19’ (cge05171, $p=8.9\times 10^{-26}$) is due to a high overlap in genes with the pathway ‘Ribosome’, as seen in Fig. S5. GO biological process and KEGG pathway enrichment of reactive genes in individual cell lines are shown in Fig. S6.

A limiting factor in the analysis of biological context by functional enrichment is the lack of reliable annotation for GO and KEGG pathway terms of most protein-coding genes in the Chinese hamster genome (4,946 annotated with GO biological process terms out of 21,841 protein-coding genes). Genes with no annotation are thus simply not considered in over-representation analysis.

To partially address this limitation we also performed over-representation analysis using the curated annotation of the secretory pathway secRecon by Masson et al. [66]. Genes that are expressed in all cell lines were enriched in secretory pathway processes including mislocalized protein degradation, pre-Golgi vesicle trafficking and co-translational translocation (Supplementary File 4). Almost all genes associated to these processes were found among the expressed gene set. Conversely, many genes that are linked to mucin-type O-glycosylation are non-expressed in all cell lines, particularly genes of the *N*-acetylgalactosamine transferase (Galnt) enzyme family. No secretory pathway processes were significantly enriched among reactive genes.

### 3.6 Distinct patterns of histone modifications distinguish expression states

Next, we explored the epigenomic context of genes that we found to be expressed, non-expressed or reactive, on the one hand to validate our findings for expressed and non-expressed protein-coding genes, and on the other hand to gain insight into the mechanisms of epigenetic control of gene expression, in particular for the reactive genes that we defined.

**Fig. 4:**
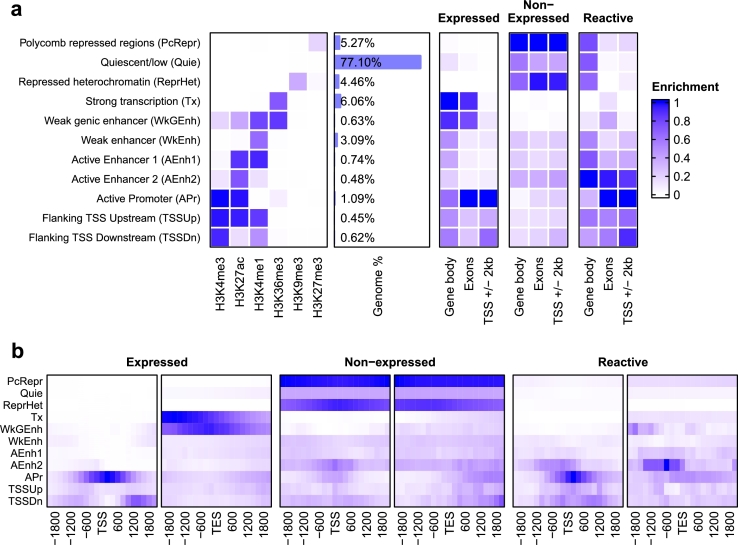
Chromatin state enrichment in gene features of different expression classes in CHO-K1. **(a)** Histone mark patterns that define corresponding chromatin states, whole-genome coverage of chromatin states, and overlap enrichment of chromatin states in three expression classes of protein-coding genes. **(b)** Neighborhood enrichment of a 2 kbp window upstream and downstream of the Transcription Start Site (TSS) and Transcription End Site (TES) of genes from different expression classes.

We used ChIP-seq data of 6 histone marks from a batch culture of CHO-K1 [17] and inferred 11 chromatin states for the PICRH genome assembly. The resulting emission parameters of the Hidden Markov Model were comparable to previously computed chromatin states for the preceeding genome assembly PICR by Rupp et al. [44] (Fig. S7). The majority of the genome's histones were unmodified across all time points under investigation with 77.10% (SD = 3.70%) of the 200 bp segments along the genome assigned to the quiescent/low chromatin state. Only 6.06% (SD = 1.50%) had histone modifications indicating strong transcription (Fig. 4a).

Gene bodies and exonic regions of expressed protein-coding genes in CHO-K1 were highly enriched in chromatin states that indicate active transcription, such as strong transcription, weak genic enhancer and active promoter (Fig. 4a). The histone modifications common in and around loci of expressed genes were therefore H3K36me3, H3k4me1, H3K4me3 and H3K27ac. Conversely, non-expressed genes resided in heterochromatic and Polycomb-repressed regions while strong transcription, weak genic enhancer and active promoter chromatin states were depleted (Fig. 4a). The enriched histone marks corresponded to H3K9me3 and H3K27me3. Reactive genes, on the other hand, exhibited chromatin state enrichment patterns of both expressed and non-expressed genes. The enrichment of the active promoter state was similar to that of expressed genes while also chromatin states such as Polycomb-repressed regions or repressed heterochromatin were enriched, although less than in non-expressed genes (Fig. 4a). This finding highlights the role of histone marks in regulating gene expression in mammalian cells.

In Fig. 4b, the enrichment of chromatin states are shown around the TSS and TES of expressed, non-expressed and reactive genes. The active promoter state was highly enriched around the TSS of expressed genes as well as histone modifications H3K4me1 and H3K4me3 that are commonly found flanking the start site. Non-expressed genes were highly enriched in chromatin states indicating repression of gene expression with Polycomb-repressed and heterochromatic regions around their TSSs and TESs. In reactive genes, the segments around putative TSSs, showed a similar pattern as expressed genes, although with less enrichment. Around their TES, the enrichment in chromatin states was indistinct compared to expressed and non-expressed genes. Notably however, no repressive states (Polycomb-repressed, repressed heterochromatin) were enriched around the TSSs and TESs. Interestingly, the active enhancer 2 state was highly enriched in segments shortly before the TES.

Indeterminate genes were previously subset to reactive genes, excluding genes that are lowly expressed around the expression threshold in all samples of a cell line. These, here called non-reactive genes, showed less enrichment of the Active Enhancer 2 chromatin state, compared to reactive genes (Fig. S8). The segments around the TSSs were similarly enriched in chromatin states as the expressed genes. At their TESs, however, the chromatin states indicating active, strong transcription were not enriched (i.e. Strong transcription, Weak genic enhancer).

Generally, all expression levels of genes were characterized by specific patterns of histone modifications and corresponding chromatin states. The enrichment of chromatin states in various features of expressed and non-expressed genes supported our findings from RNA-seq data. Non-expressed genes reside in heterochromatic, condensed regions of the genome while transcription of expressed genes is facilitated by histone marks which open up the chromatin. Reactive genes exhibit a much vaguer picture in terms of chromatin state patterns with enrichment values similar to both expressed and non-expressed genes. The ChIP-seq data from which the chromatin states were defined were only from one batch experiment of a single cell line, in contrast to multiple RNA-seq datasets and experiments in our study. Therefore, with the data currently available, the dynamics of epigenetic control of various culture conditions and phenotypes as reflected by chromatin states in the corresponding genome regions, cannot be fully analyzed. However, since the ON/OFF pattern of gene expression is highly consistent, with more than 9000 genes consistently expressed in all CHO samples irrespective of condition or lineage, we hypothesize that chromatin states in the corresponding genomic regions are likely to be equally consistent.

## 4 Conclusions

In this study we present a comprehensive meta-analysis of CHO transcriptomes. Although RNA-seq is a routinely used analysis, to the best of our knowledge, no attempt to unify and compare samples from different studies has been done before for CHO cells. After a thorough study of the best methodologies to normalize and compare experiments performed under highly heterogeneous conditions, we found that expressed and non-expressed genes are highly concordant between cell lines and conditions. This suggests that the major differences between cell lines and culture conditions, seen in our exploratory data analysis, stem primarily from the precise regulation of gene expression levels for each active gene. This underscores the necessity for novel genetic engineering tools that provide a fine-tuned control of the expression level in order to improve attributes of cell lines [9], [10]. Our findings further unveil a set of genes that adapts to different conditions by major shifts in expression level, ranging from near-threshold or off to active transcription. These are primarily, and not surprisingly, connected to stress response mechanisms. Further investigations are needed to unravel the dynamics of the CHO transcriptome across various culture conditions and phenotypes, in order to gain a deeper, more comprehensive understanding of fundamental intracellular mechanisms. These insights can lay the foundation for significant improvements in terms of CHO's role as the main cell factory for biopharmaceutical products by facilitating rational engineering of cellular characteristics and better prediction of phenotypic behavior, for instance in subclones during cell line development.

## CRediT authorship contribution statement

**Markus Riedl:** Writing – review & editing, Writing – original draft, Visualization, Validation, Software, Methodology, Formal analysis, Conceptualization. **Caterina Ruggeri:** Writing – review & editing, Software, Methodology, Data curation, Conceptualization. **Nicolas Marx:** Writing – review & editing, Supervision, Conceptualization. **Nicole Borth:** Writing – review & editing, Supervision, Resources, Project administration, Funding acquisition, Conceptualization.

## Declaration of Competing Interest

The authors declare that they have no known competing financial interests or personal relationships that could have appeared to influence the work reported in this paper.

## Data Availability

Code for reproducing analyses and figures is available at github.com/NBorthLab/CHO-coding-transcriptome. RNA-seq and ChIP-seq data are visualized in a genome browser available at cgr-referencegenome.boku.ac.at/new.

## Appendix A Supplementary material

MMC 1 Supplementary figures and tables. MMC 1

MMC 2 Details and metadata of all included datasets. MMC 2

MMC 3 Gene expression classifications in CHO-K1, CHO-S and CHO-DXB11. MMC 3

MMC 4 Enriched secRecon terms. MMC 4
