# Supplementary material for: Fantastic genes and where to find them expressed in CHO
Source: Comput Struct Biotechnol J. 2025 Apr 2;27:1407–15. doi: 10.1016/j.csbj.2025.03.050 (PMC12002940; doi:10.1016/j.csbj.2025.03.050)
Supplement: MMC 1 — Supplementary figures and tables. [file mmc1.pdf]

1 Supplementary Figures

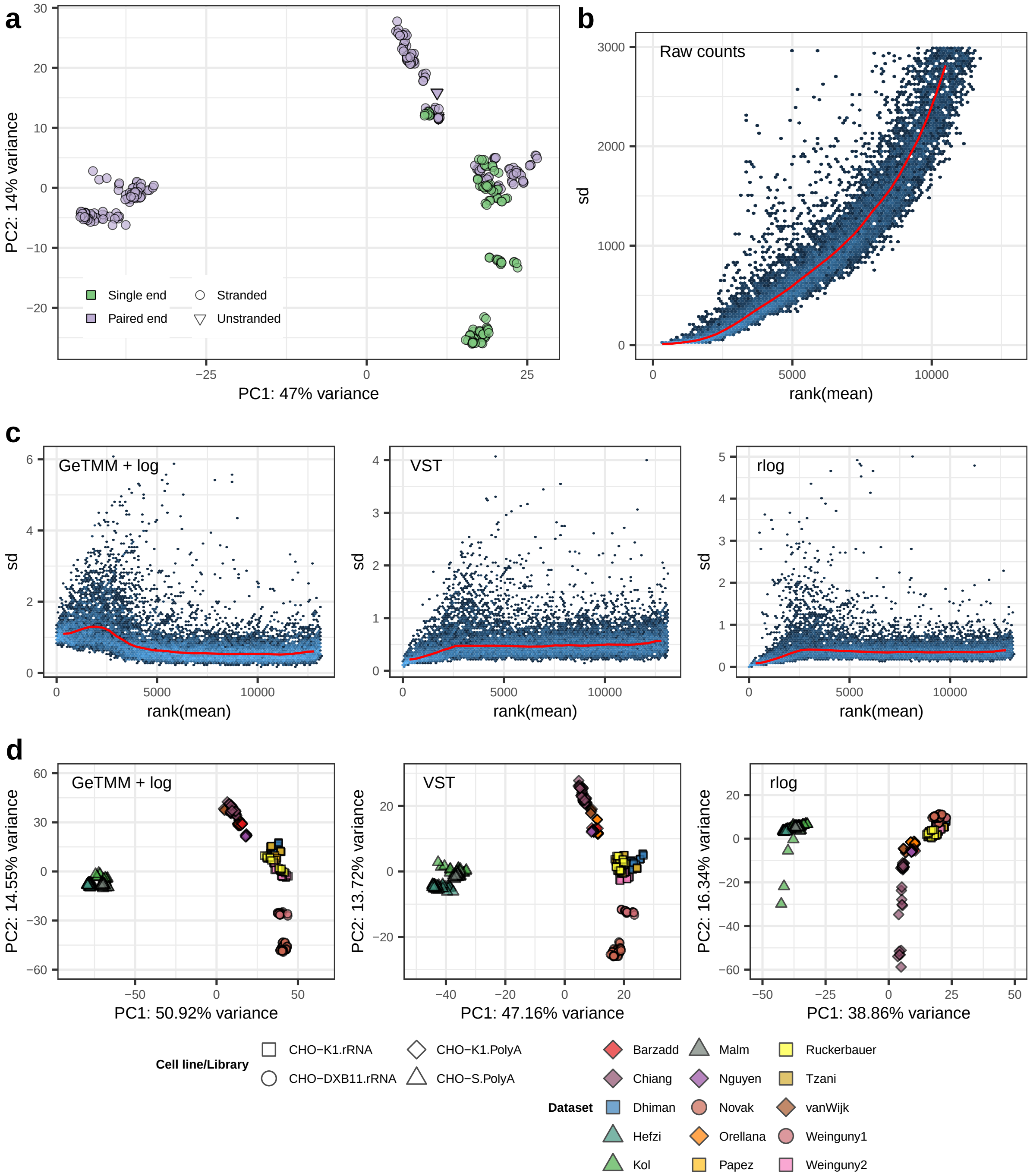

Supplementary Figure S1: Data exploration by dimension reduction using different normalization and transformation techniques. **(a)** Principal component analysis following variance-stabilizing transformation indicating the use of single- and paired-end sequencing techniques as well as stranded and unstranded RNA-sequencing libraries. **(b)** Mean-SD plot of untransformed, raw gene counts. **(c)** Mean-SD plots of transformed counts using different normalization and transformation techniques. **(d)** Principal component analysis following various normalization and transformation techniques. Colors indicate the dataset, shapes indicate the cell line and library preparation method poly(A) enrichment or rRNA depletion. PC, Principal Component; GeTMM, Gene-length corrected Trimmed Means of M-values; VST, Variance-Stabilizing Transformation; rlog, regularized logarithm transformation;

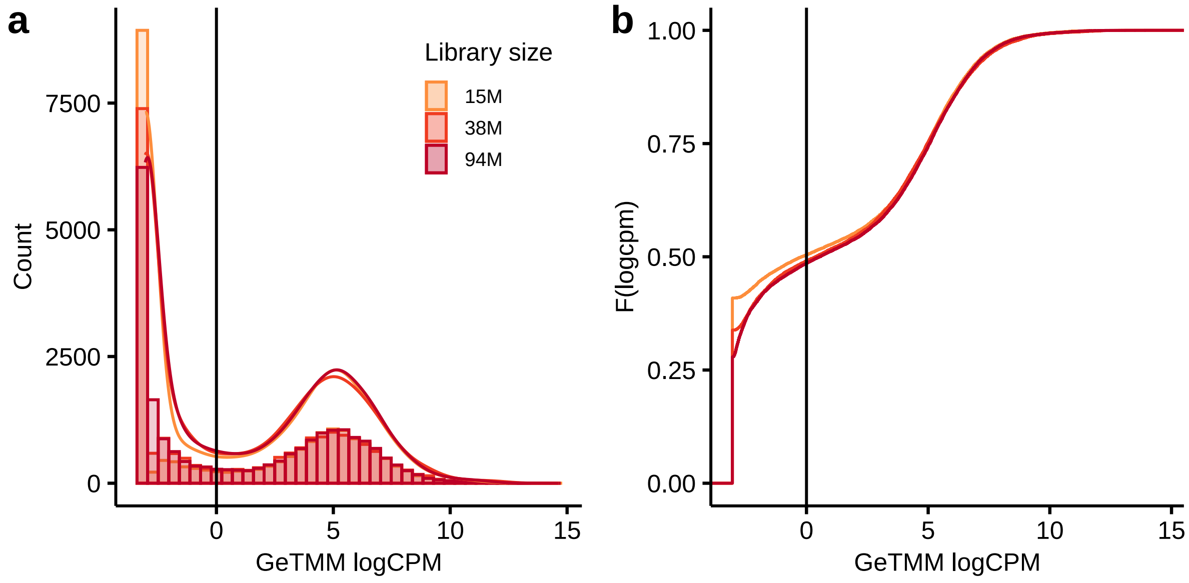

Supplementary Figure S2: Distribution of normalized gene expressions using GeTMM (Gene-length corrected Trimmed Means of M-values). Three samples with different library sizes are shown as **(a)** histogram and **(b)** cumulative density. Vertical line at logCPM = 0 indicates the expression threshold used for classifying genes according to expression.

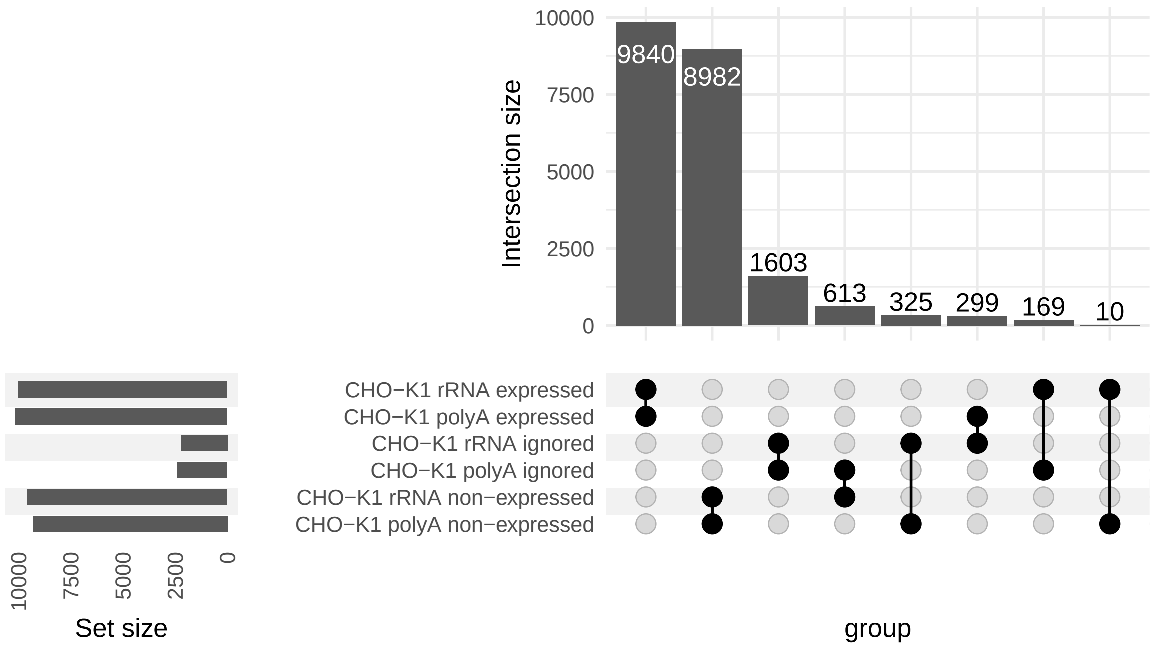

Supplementary Figure S3: Upset plot showing the set sizes and intersections of expression, indeterminate and non-expressed gene sets in CHO-K1 samples employing different library preparation methods.

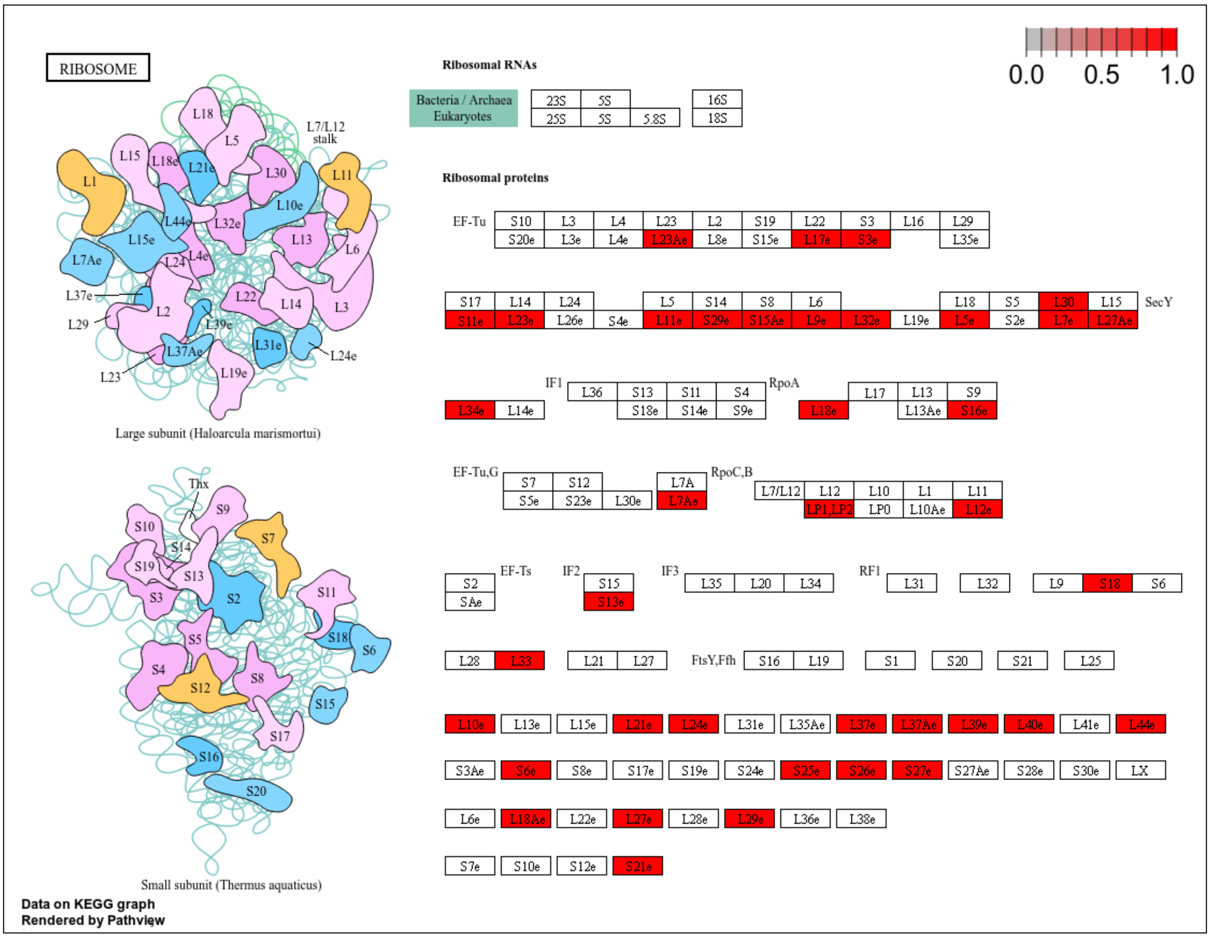

Supplementary Figure S4: Reactive genes in the KEGG pathway 'Ribosome' (cge03010). Ribosomal proteins and ribosomeal like proteins that are colored in red are part of the reactive gene set in at least one of the three cell lines: CHO-K1, CHO-S or CHO-DXB11.

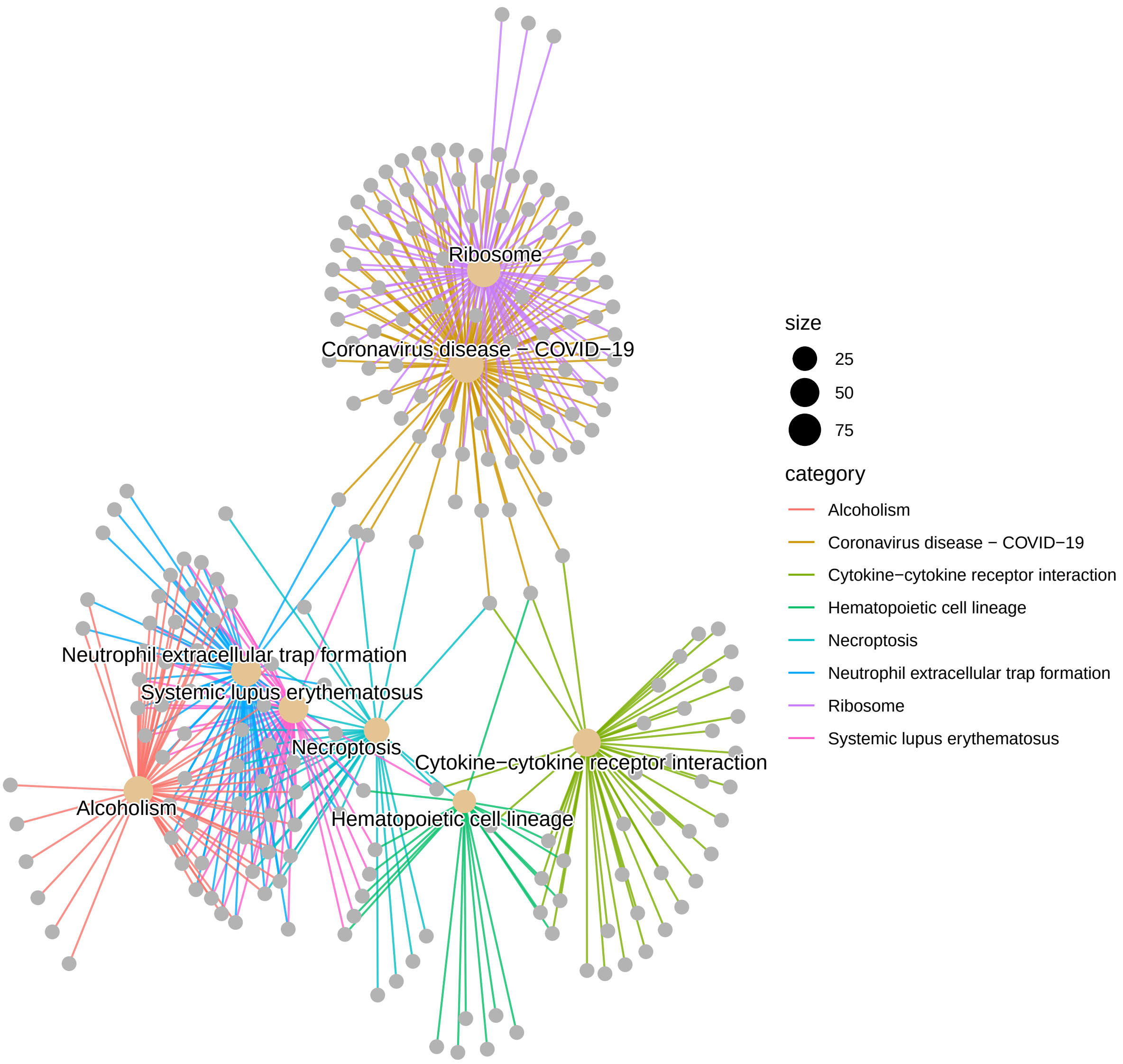

Supplementary Figure S5: Gene-concept network plot of enriched KEGG pathway terms in reactive genes. Colored nodes are KEGG pathway terms, grey nodes are genes. Edges connect genes to associated KEGG pathway terms.

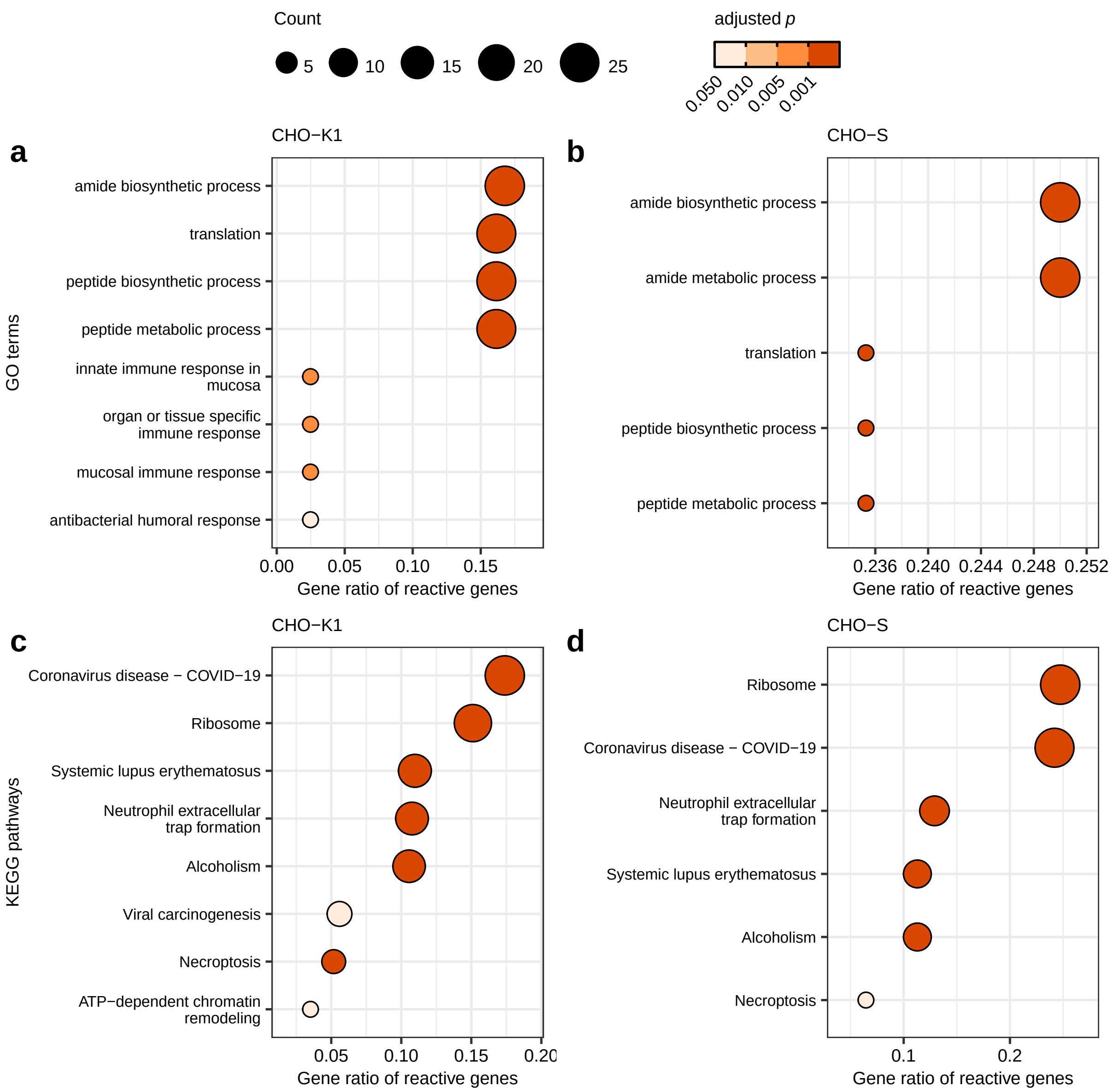

Supplementary Figure S6: Functional enrichment of reactive genes in CHO cell lines. GO biological process terms enriched in **(a)** CHO-K1 and **(c)** CHO-S as well as KEGG pathway enrichment of **(c)** CHO-K1 and **(d)** CHO-S. Not shown is the enrichment of one KEGG pathway term in reactive genes of CHO-DXB11: Cytokine-cytokine receptor interaction (gene ratio 14/128; adjusted $p = 0.00522$). No GO biological process terms were enriched among reactive genes of CHO-DXB11.

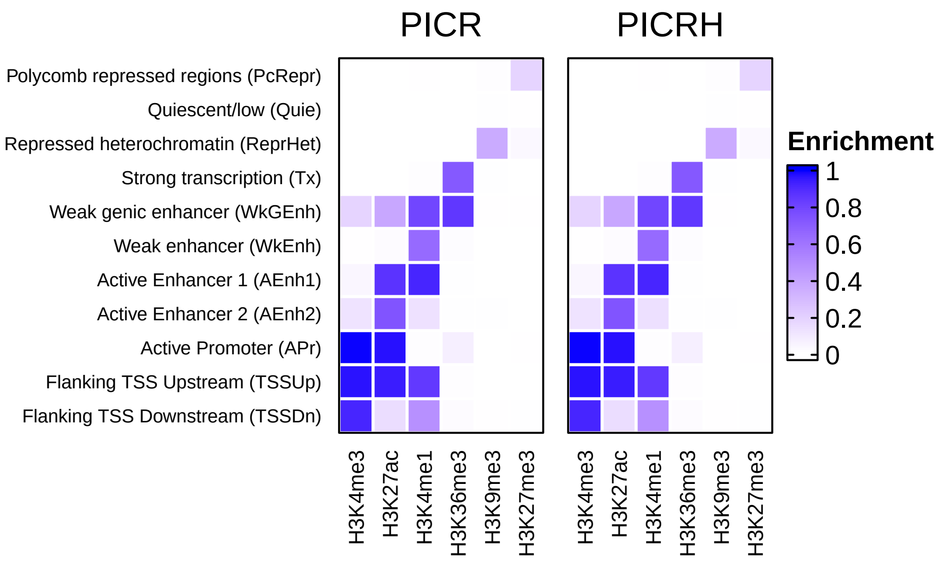

Supplementary Figure S7: Hidden Markov model emission parameters for 11 chromatin states based on 6 histone marks. The emission parameters from the model for the PICR genome assembly by Rupp et al [1] (left) are compared with the model parameters for the current PICRH genome assembly [2] (right).

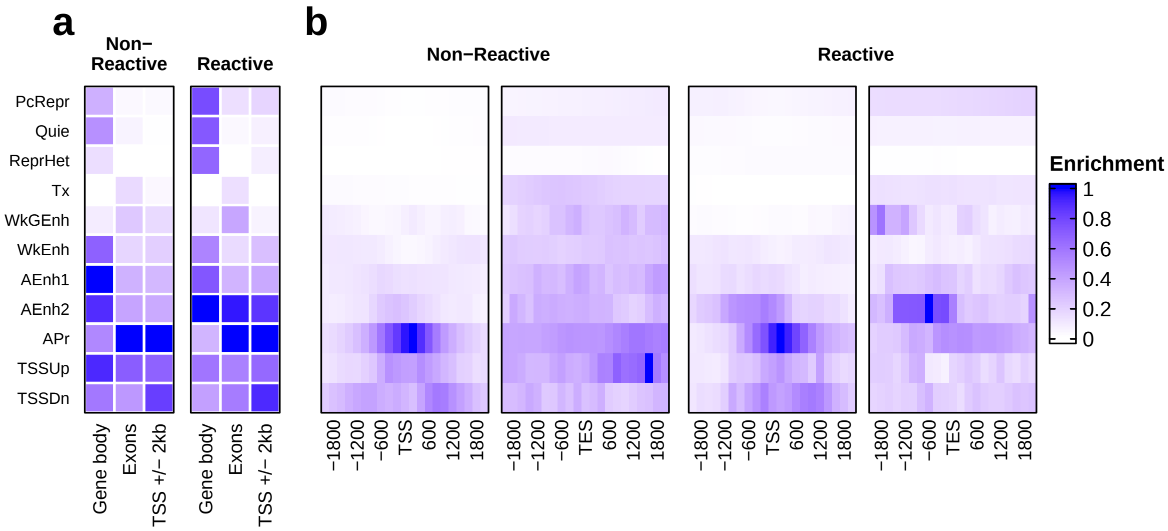

Supplementary Figure S8: Chromatin states enrichments in gene features of reactive and non-reactive genes. **(a)** Overlap enrichment in gene bodies, exons and around the transcription start site of non-reactive (left) and reactive genes (right). **(b)** Neighborhood enrichment in 200 bp segments around the transcriptions start sites and end sites of non-reactive (left) and reactive genes (right). TSS, Transcription Start Site; TES, Transcription End Site; PcRepr, Polycomb-repressed regions; Quie, Quiescent/low; ReprHet, Repressed heterochromatin; Tx, Strong Transcription; WkGEnh, Weak genic enhancer; WkEnh, Weak enhancer; AEnh1, Active Enhancer 1; AEnh2, Active Enhancer 2; APr, Active Promoter; TSSUp, Flanking TSS Upstream; TSSDn, Flanking TSS Downstream;

2 Supplementary Tables

Supplementary Table S1: Enriched biological process gene ontology terms in gene sets that are differentially expressed (expressed vs non-expressed in cell lines) or exclusively expressed in a cell line. GeneRatio refers to genes in the gene set; BgRatio refer to the ratio in the ontology tree. Multiple testing correction was conducted using the Benjamini-Hochberg method.

ID	Description	GeneRatio	BgRatio	adjusted p	geneID
K1 vs S					
GO:1902033	regulation of hematopoietic stem cell proliferation	3/26	4/4946	4.21×10^{-4}	Kitlg/Snai2/Eif2ak2
GO:0048525	negative regulation of viral process	3/26	18/4946	1.85×10^{-2}	Ptx3/Ifih1/Eif2ak2
GO:0072091	regulation of stem cell proliferation	3/26	18/4946	1.85×10^{-2}	Kitlg/Snai2/Eif2ak2
GO:0033033	negative regulation of myeloid cell apoptotic process	2/26	4/4946	1.85×10^{-2}	Kitlg/Snai2
GO:0150146	cell junction disassembly	2/26	4/4946	1.85×10^{-2}	Snai2/Ston1
GO:1901532	regulation of hematopoietic progenitor cell differentiation	2/26	4/4946	1.85×10^{-2}	Kitlg/Eif2ak2
GO:0072089	stem cell proliferation	3/26	24/4946	2.39×10^{-2}	Kitlg/Snai2/Eif2ak2
GO:0006929	substrate-dependent cell migration	2/26	7/4946	3.74×10^{-2}	Snai2/Ston1
GO:0060317	cardiac epithelial to mesenchymal transition	2/26	8/4946	3.86×10^{-2}	Snai2/Emp2
GO:0048863	stem cell differentiation	3/26	35/4946	3.86×10^{-2}	Kitlg/Snai2/Eif2ak2
GO:0030100	regulation of endocytosis	3/26	37/4946	4.14×10^{-2}	Ptx3/Sfrp4/Ston1
GO:0033028	myeloid cell apoptotic process	2/26	9/4946	4.14×10^{-2}	Kitlg/Snai2
GO:0048762	mesenchymal cell differentiation	3/26	39/4946	4.14×10^{-2}	Kitlg/Snai2/Emp2
GO:0014032	neural crest cell development	2/26	10/4946	4.14×10^{-2}	Kitlg/Snai2
GO:0140374	antiviral innate immune response	2/26	10/4946	4.14×10^{-2}	Ifih1/Eif2ak2
GO:0019058	viral life cycle	3/26	46/4946	4.96×10^{-2}	Ptx3/Ifih1/Eif2ak2
GO:0032602	chemokine production	2/26	12/4946	4.96×10^{-2}	Snai2/Eif2ak2
GO:0032642	regulation of chemokine production	2/26	12/4946	4.96×10^{-2}	Snai2/Eif2ak2
DXB11 vs K1					
GO:0009395	phospholipid catabolic process	1/1	10/4946	2.22×10^{-2}	Plcg2
GO:0046434	organophosphate catabolic process	1/1	31/4946	3.45×10^{-2}	Plcg2

Supplementary Table S1: *(continued)*

ID	Description	GeneRatio	BgRatio	adjusted p	geneID
K1 vs DXB11					
GO:0033688	regulation of osteoblast proliferation	1/2	4/4946	3.95×10^{-2}	Eif2ak2
GO:0035455	response to interferon-alpha	1/2	4/4946	3.95×10^{-2}	Eif2ak2
GO:1901532	regulation of hematopoietic progenitor cell differentiation	1/2	4/4946	3.95×10^{-2}	Eif2ak2
GO:1902033	regulation of hematopoietic stem cell proliferation	1/2	4/4946	3.95×10^{-2}	Eif2ak2
GO:0033687	osteoblast proliferation	1/2	5/4946	3.95×10^{-2}	Eif2ak2
GO:0060218	hematopoietic stem cell differentiation	1/2	5/4946	3.95×10^{-2}	Eif2ak2
GO:0032722	positive regulation of chemokine production	1/2	8/4946	3.95×10^{-2}	Eif2ak2
GO:0071425	hematopoietic stem cell proliferation	1/2	8/4946	3.95×10^{-2}	Eif2ak2
GO:0044546	NLRP3 inflammasome complex assembly	1/2	9/4946	3.95×10^{-2}	Eif2ak2
GO:0140632	canonical inflammasome complex assembly	1/2	9/4946	3.95×10^{-2}	Eif2ak2
GO:0141084	inflammasome-mediated signaling pathway	1/2	9/4946	3.95×10^{-2}	Eif2ak2
GO:0141085	regulation of inflammasome-mediated signaling pathway	1/2	9/4946	3.95×10^{-2}	Eif2ak2
GO:1900225	regulation of NLRP3 inflammasome complex assembly	1/2	9/4946	3.95×10^{-2}	Eif2ak2
GO:2000736	regulation of stem cell differentiation	1/2	9/4946	3.95×10^{-2}	Eif2ak2
GO:0045071	negative regulation of viral genome replication	1/2	10/4946	3.95×10^{-2}	Eif2ak2
GO:0140374	antiviral innate immune response	1/2	10/4946	3.95×10^{-2}	Eif2ak2
GO:0034198	cellular response to amino acid starvation	1/2	11/4946	3.95×10^{-2}	Eif2ak2
GO:0032602	chemokine production	1/2	12/4946	3.95×10^{-2}	Eif2ak2

Supplementary Table S1: *(continued)*

ID	Description	GeneRatio	BgRatio	adjusted p	geneID
GO:0032642	regulation of chemokine production	1/2	12/4946	3.95×10^{-2}	Eif2ak2
GO:1990928	response to amino acid starvation	1/2	12/4946	3.95×10^{-2}	Eif2ak2
GO:1901224	positive regulation of non-canonical NF-kappaB signal transduction	1/2	15/4946	4.38×10^{-2}	Eif2ak2
GO:0030968	endoplasmic reticulum unfolded protein response	1/2	18/4946	4.38×10^{-2}	Eif2ak2
GO:0039531	regulation of viral-induced cytoplasmic pattern recognition receptor signaling pathway	1/2	18/4946	4.38×10^{-2}	Eif2ak2
GO:0072091	regulation of stem cell proliferation	1/2	18/4946	4.38×10^{-2}	Eif2ak2
GO:1901222	regulation of non-canonical NF-kappaB signal transduction	1/2	20/4946	4.38×10^{-2}	Eif2ak2
GO:0034620	cellular response to unfolded protein	1/2	21/4946	4.38×10^{-2}	Eif2ak2
GO:0002753	cytosolic pattern recognition receptor signaling pathway	1/2	22/4946	4.38×10^{-2}	Eif2ak2
GO:0032874	positive regulation of stress-activated MAPK cascade	1/2	22/4946	4.38×10^{-2}	Eif2ak2
GO:0035967	cellular response to topologically incorrect protein	1/2	23/4946	4.38×10^{-2}	Eif2ak2
GO:0070304	positive regulation of stress-activated protein kinase signaling cascade	1/2	23/4946	4.38×10^{-2}	Eif2ak2
GO:0046777	protein autophosphorylation	1/2	24/4946	4.38×10^{-2}	Eif2ak2
GO:0062207	regulation of pattern recognition receptor signaling pathway	1/2	24/4946	4.38×10^{-2}	Eif2ak2
GO:0072089	stem cell proliferation	1/2	24/4946	4.38×10^{-2}	Eif2ak2
GO:0019079	viral genome replication	1/2	25/4946	4.44×10^{-2}	Eif2ak2
GO:0006986	response to unfolded protein	1/2	28/4946	4.54×10^{-2}	Eif2ak2
GO:0051092	positive regulation of NF-kappaB transcription factor activity	1/2	29/4946	4.54×10^{-2}	Eif2ak2

Supplementary Table S1: *(continued)*

ID	Description	GeneRatio	BgRatio	adjusted p	geneID
GO:0017148	negative regulation of translation	1/2	32/4946	4.67×10^{-2}	Eif2ak2
GO:0048863	stem cell differentiation	1/2	35/4946	4.67×10^{-2}	Eif2ak2
GO:0002758	innate immune response-activating signaling pathway	1/2	37/4946	4.67×10^{-2}	Eif2ak2
GO:0034249	negative regulation of amide metabolic process	1/2	37/4946	4.67×10^{-2}	Eif2ak2
only DXB11					
GO:0002227	innate immune response in mucosa	3/70	5/4946	1.51×10^{-2}	LOC100772735/LOC100751440/LOC100751148
GO:0002251	organ or tissue specific immune response	3/70	6/4946	1.51×10^{-2}	LOC100772735/LOC100751440/LOC100751148
GO:0002385	mucosal immune response	3/70	6/4946	1.51×10^{-2}	LOC100772735/LOC100751440/LOC100751148
GO:0019731	antibacterial humoral response	3/70	8/4946	3.11×10^{-2}	LOC100772735/LOC100751440/LOC100751148

References

[1] O. Rupp, M. L. MacDonald, S. Li, H. Dhiman, S. Polson, S. Griep, K. Heffner, I. Hernandez, K. Brinkrolf, V. Jadhav, M. Samoudi, H. Hao, B. Kingham, A. Goesmann, M. J. Betenbaugh, N. E. Lewis, N. Borth, K. H. Lee, A reference genome of the Chinese hamster based on a hybrid assembly strategy, Biotechnology and Bioengineering 115 (2018) 2087–2100. doi:10.1002/bit.26722.

[2] W. Hilliard, M. L. MacDonald, K. H. Lee, Chromosome-scale scaffolds for the Chinese hamster reference genome assembly to facilitate the study of the CHO epigenome, Biotechnology and Bioengineering 117 (2020) 2331–2339. doi:10.1002/bit.27432.
